# Resistance exercising on unstable surface leads to Pupil Dilation

**DOI:** 10.1186/s13102-024-00858-w

**Published:** 2024-03-04

**Authors:** Lisa Claußen, Tabea Heidelbach

**Affiliations:** Institute of Sports and Sport Science, University of Kassel, Kassel, Germany

**Keywords:** Metastable resistance training, Surface instability, Task complexity, Pupilometry, Pupil size, Mental effort

## Abstract

**Background:**

Chronic resistance training and acute resistance exercises improve physical performance and can enhance cognitive performance. However, there is still uncertainty about the mechanism(s) responsible for cognitive improvement following resistance training and exercise. Recent findings suggest that resistance exercise has metabolic as well as cognitive demands, which potentially activate similar neural circuitry associated with higher-order cognitive function tasks. Exercising on unstable devices increases the coordinative and metabolic demands and thus may further increase cognitive activation during resistance exercise. The measurement of pupil diameter could provide indications of cognitive activation and arousal during resistance exercise. Pupil dilation is linked to the activity in multiple neuromodulatory systems (e.g., activation of the locus coeruleus and the release of the neurotransmitter norepinephrine (LC-NE system)), which are involved in supporting processes for executive control. Therefore, the purpose of this study was to compare the cognitive activation measured by pupil diameter during an acute bout of resistance exercise on stable and unstable surfaces.

**Methods:**

18 participants (23.5 ± 1.5 years; 10 females) performed ten kettlebell squats in a preferred repetition velocity in stable and unstable (BOSU® Balance Trainer) ground conditions. Pupil diameter was recorded with eye tracking glasses (SMI ETG) during standing (baseline) and during squatting. Raw pupil data were cleaned of artifacts (missing values were linearly interpolated) and subjected to a subtractive baseline correction. A student t-test was used to compare mean pupil diameter between ground conditions.

**Results:**

The mean pupil diameter was significantly greater during squats in the unstable condition than in the stable condition, *t* (17) = -2.63, *p* =.018, Cohen’s *d*_Z_ = -0.62; stable: 0.49 ± 0.32 mm; unstable: 0.61 ± 0.25 mm).

**Conclusion:**

As indicated by pupil dilation, the use of unstable devices can increase the cognitive activation and effort during acute bouts of resistance exercise. Since pupil dilation is only an indirect method, further investigations are necessary to describe causes and effects of neuromodulatory system activity during resistance exercise. Resistance training with and without surface instability can be recommended to people of all ages as a physically and cognitively challenging training program contributing to the preservation of both physical and cognitive functioning.

## Background

Chronic resistance training and acute resistance exercises improve physical performance and can induce structural and functional changes in the brain associated with enhanced cognitive performance [1, 2]. Resistance training is recommended for people of all ages [3] as it can contribute to quality of life, especially in older adults, by mitigating age-related physical (e.g., muscle strength) and cognitive decline (e.g., executive functions (EF)) [4].

In the debate on training programs for improving cognitive functioning, there has been discussion about the effectiveness of “mindless” programs [5, 6]. However, chronic metastable resistance training, which involves resistance exercises on unstable surface, has shown promise in improving both physical and cognitive performance [7]. These exercises require coordination and cognitive effort due to increased vertical and transversal ground reaction forces [8] and demands on visual perception [9], which might involve a different mode of information processing [10]. Cognitive demand refers to “the amount of mental demand a task puts on mental resources” [11]. Cognitively demanding tasks require mental effort, processing, and resources to be executed within a limited time and/or with a certain level of performance [11]. In the field of exercise science, cognitively demanding refers to physical exercises that are complex and novel, requiring whole-body coordination and sustained processing of sensorimotor information [12, 13].

Recent literature suggests that cognitively demanding training interventions have a greater effect on cognitive functioning than resistance training interventions alone [14]. According to the “cognitive stimulation hypothesis”, complex and novel motor tasks are assumed to activate higher-order cognitive processes and include learning processes, resulting in task-specific improvements in cognitive performance [15–17]. Consistent with this approach, Eckardt and colleagues [18] found improvements in cognitive performance in older adults who executed resistance training on unstable surfaces compared to older adults who completed a machine-based resistance-training program. They attributed their findings to increased cognitive demands during metastable resistance training, partly due to increased demands on sensorimotor and postural control, and partly due to the novelty of unstable surfaces for older adults [7].

Currently, there is still uncertainty about the mechanism(s) underlying the relationship between chronic resistance training and cognition [19, 20]. On one hand, acute and chronic resistance training can induce changes in physiological biomarkers (e.g., catecholamines, insulin-like growth factor-1 (IGF-1), brain-derived neurotrophic factor) due to the intensity of repeated movements [21], which are summarized in the “neurothropic hypothesis” [22–24]. These changes trigger neurobiological processes that can influence global neuroplasticity and, at best, improve cognitive functions [21, 25, 26]. On the other hand, cognitively demanding exercises are thought to activate similar neural circuitry associated with EF tasks during an acute bout of exercise [12, 15]. The same hypothesis could also apply to the effects of resistance training and is supported by the results of Herold, Hamacher and colleagues [27], who showed that acute free-weight resistance exercising requires attentional resources. Both exercise intensity and motor task complexity are associated with acute changes in brain activation and arousal [11, 28].

As suggested by Herold, Törpel and colleagues [1], research on the acute effects of resistance exercise may help to identify relevant training and exercise variables which contribute to cognitive improvements. Therefore, the purpose of this study is to investigate cognitive activation during resistance exercise on stable and unstable surfaces in a population of young adults.

The neurophysiological component involved in arousal and activation is the reticular activation system, which consists of several interconnected arousal systems. They are distinguished based on their specific neurotransmitters and neuromodulation: the noradrenergic, dopaminergic, and serotonergic systems. Animal and human studies show that these systems respond to acute physical stress and release catecholamines (norepinephrine and dopamine) and indolamines (serotonin or 5-hydroxytryptamine) in the brain. Many of the acute and chronic effects of physical exercise on cognitive processes are closely related to catecholaminergic and indolaminergic neuromodulation of neuronal networks involved in information processing [29].

The activation of the reticular activation system is linked to pupil size and can be measured by pupillometry [11, 30]. Pupillometry measures pupil size and uses changes in size to assess states of arousal and attention [31]. It also serves as an indicator of physical and mental effort [32]. An increase in pupil size indicates activation of the sympathetic and inhibition of the parasympathetic part of the autonomic nervous system and is associated with activity in multiple brain regions and neuromodulatory systems, including the medial prefrontal cortex, the inferior colliculus, the cholinergic system, and the locus coeruleus norepiphrine system (LC-NE system) [33, 34]. The locus coeruleus (LC) is a small nucleus located in the brainstem and is part of the reticular activation system. It releases the neurotransmitter norepinephrine (NE) and can innervate large parts of the brain, including the cerebral cortex, thalamus, hypothalamus, cerebellum, midbrain, and spinal cord [35]. An increase in pupil diameter indicates the inhibitory effect of LC on the parasympathetic Edinger Westphal nucleus. The sphincter pupillae muscle, which constricts the pupil, receives efferent fibers from this nucleus. Furthermore, LC activity activates the sympathetic fibers that innervate the dilator pupillae muscle [36–38]. In addition to changes in pupil diameter, the activity of the LC or LC-NE system is related to the regulation of attention and the filtering of incoming information [39]. The release of NE promotes alertness and sensory gating in the auditory and sensory cortex. Sensory gating describes a way of information processing in which specific stimuli are processed while task-irrelevant stimuli are suppressed [40]. Similar gating effects have been found in other brain areas associated with the LC [38, 41, 42]. In the frontal areas of the brain, this filtering function affects the size of the attentional focus and thus, underlies many complex cognitive functions such as working memory, learning, and decision-making [38, 43]. However, pupil diameter can only accurately predict a small fraction of LC activity because the relationship between changes in pupil size and LC activity is influenced by brain states [33].

Nevertheless, pupil dilation has previously been observed with increasing task difficulty in cognitive tasks [44, 45]. Studies of motor tasks also showed changes in pupil size as a function of task difficulty [46–49]. Moreover, studies of upright standing with varying balance demands showed that mental effort as measured by pupil size increased with heightened demands for postural control [50, 51]. In addition, there appears to be a positive correlation between the intensity of physical exercise and pupil dilation, indicating an increase in arousal in an intensity-dependent manner [52, 53].

While there is evidence that resistance exercises with free weights are metabolically and cognitively demanding [27], the use of unstable devices may further increase effort and cognitive activation during exercise due to task complexity, higher energy costs and higher rate of perceived exertion [54].

However, cognitive activation during resistance exercise on stable and unstable surfaces has not yet been analyzed. Therefore, this study aims to investigate the cognitive activation measured by pupil diameter during acute resistance exercises and to compare the influence of stable and unstable ground conditions. Consequently, the investigation of the acute effects of exercising on unstable devices may contribute to the understanding of how chronic resistance training with surface instability may enhance EF [7, 18]. It is hypothesized that pupil diameter will be larger during squats on unstable ground conditions compared to stable ground conditions due to increased metabolic and cognitive demands with increasing surface instability.

## Materials and methods

### Power analysis

For the power analysis, we focused on the main dependent variable: the pupil diameter. Saeedpour-Parizi et al. [48] and Kahya et al. [51] showed large effect sizes for differences between task conditions (d > 0.9). We assumed a medium-to-large effect size (d > 0.7) for the power analysis of a dependent t-test with a Type I error rate of α = 0.05 for two-sided testing and set the test power at 0.80. The power analysis revealed that 18 participants are required.

### Participants

Eighteen healthy young adults (f = 10) with an average age of 23.5 years (*SD* = 1.5) participated in the two sessions of this study. All subjects were regularly involved in sporting activities and had not previously received refractive treatment to correct visual impairment. This was important because such surgery can affect pupil dilatation and could have biased the measurement [55]. In addition, the participants confirmed that they did not haveany musculoskeletal or cardiovascular impairments, as well as no neurological or psychiatric diseases, through self-reports and the German version of the Physical Activity Readiness Questionnaire (PAR-Q) [56]. Participants who answered “yes” to two or more questions of the PAR-Q, which indicates an increased health risk during physical exercise, were excluded. All participants were informed about the study procedure at the beginning and gave their written consent to participate. The study procedures were in accordance with the Declaration of Helsinki (1964) and were approved by the local ethics committee of the University of Kassel (E05202004).

### Procedure

**Fig. 1:**
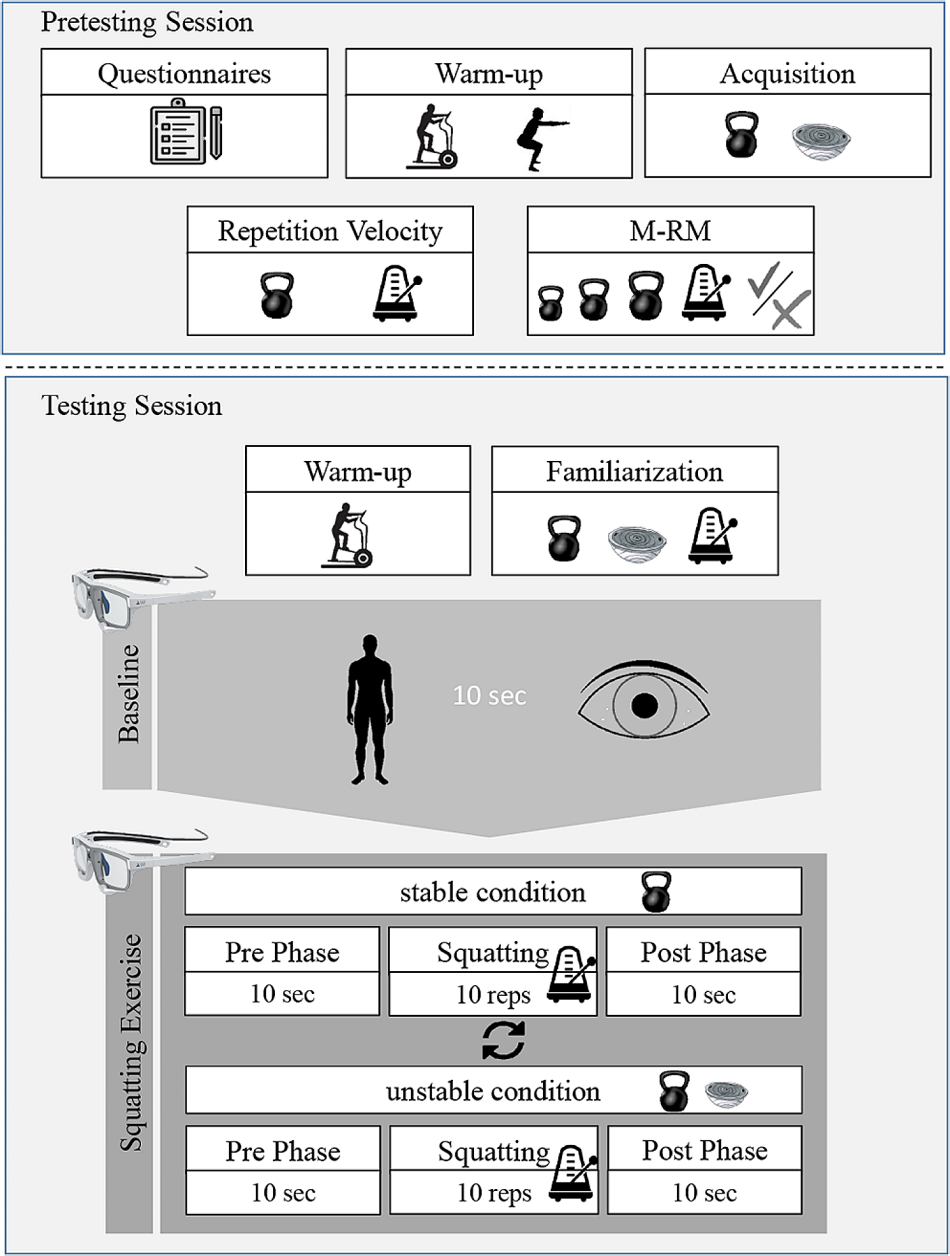
Study procedure with pretesting and testing session

The procedure of this study was adapted to the study protocol of Herold et al. [27]. Investigations were conducted at the Sport Institute of the University of Kassel and consisted of a pretesting and testing session, taking place at least 48 h apart. The procedure is illustrated in Fig. 1.

### Pretesting session

In order to identify sample characteristics and factors of influence, participants were asked questions on sport behavior as well as on experience with resistance training and instability. In the first session, participants answered a physical activity questionnaire (BSA; [57]) which assessed the physical activity and sport exercise in the last four weeks. Additionally, participants had to rate their experience in resistance training on a scale from 1 to 10 (1 = no experience; 10 = much experience) as well as indicate how often they used unstable devices while exercising (1 = never, 5 = always). Furthermore, all participants completed the eleven items of the Montreal Cognitive Assessment (MoCA) [58] in order to exclude cognitive impairments.

Subsequently, participants completed a five-minute workout on a cross trainer (1 W per kg body weight) for a general warm-up of both legs and arms. For the specific warm-up, participants were asked to select a kettlebell which they could easily lift for ten repetitions of the squatting exercise on stable (flat floor) and unstable (BOSU® Balance Trainer; flat side facing up) ground conditions. Participants were instructed to perform squats at a preferred pace, with a proper technique (from a shoulder width standing position marked on the floor and on the Bosu® Balance Trainer for orientationand horizontal thighs as the optimal squath depth) and with holding a kettlebell at chest height. The kettlebells were chosen as free weights as they are commonly used for performing squats with weight held in front of the chest and moreover, do not interfere with the cable connection of the eye-tracking system used to capture pupil data. The experimenter gave feedback and announced the last repetition of the set, thus participants did not have to count their repetitions. The familiarization phase to the unstable device was repeated if participants were not able to keep balance or felt insecure.

In order to control for time under tension (TuT) across the different ground conditions [8] and to allow for the habitual pace while squatting, an individual repetition velocity was defined for each participant. First, participants’ individual repetition velocity was determined for both ground conditions separately by using a Metronome-App (The Metronome by Soundbrenner, version 1.24 for Android) on a Samsung Galaxy Tab A6 and subsequently averaged across conditions. The individual repetition velocity was determined by tapping on the screen when participants repeatedly reached the deepest squatting position and the standing position in a set of ten repetitions. Accordingly, the metronome calculated the beats per minute (BPM) and provided different sounds for each turning point. With the averaged repetition velocity, the participants performed a familiarization session consisting of ten repetitions each on stable and unstable ground. After completion, they were asked to rate how they could manage the kettlebell squats with the fixed individual repetition velocity on a 11-point feeling scale (FS) ranging from − 5 (very bad) to + 5 (very good). If participants indicated 0 (neutral) or worse in either the stable or the unstable condition, the average BPM were increased or decreased by 5% and tested again in both ground conditions in order to exclude stress responses due to time pressure.

In addition, an inertial measurement unit (IMU) (Vmaxpro, version 6.2.6, Magdeburg, Germany) attached to the kettlebell was used to monitor the vertical displacement and the movement velocity during squat performance. The sensor was controlled via an iPad 6 with the Vmaxpro App (version 4.2.1). Participants received feedback on their squatting performance if kettlebell squats differed between conditions.

The final stage of the pretesting session comprised a multiple repetition maximum (M-RM) test on stable ground in order to adjust the additional weights to individual abilities. An M-RM was preferred to a one-repetition maximum (1-RM) in this study as the kettlebells were held without a safety device and lifting the maximum weight increases risk of muscular injury, especially for individuals not used to weight training [59]. For the M-RM testing, participants were instructed to perform as many squats as they could manage with a self-selected starting weight in their previously determined individual repetition velocity on the flat floor. If participants reached ten repetitions, the current set ended. Participants were asked to rate their perceived exertion and their repetitions in reserve (RIR) [60]. After a three-minute pause, the kettlebell weight was increased by four kilograms and participants once again performed as many repetitions as possible with the current kettlebell weight. This procedure continued until either the participants conducted a set of less than ten repetitions without any RIR or there was no heavier kettlebell (max. 40 kg) available. If participants were able to lift the 40 kg kettlebell during M-RT, the set ended when they were no longer able to perform kettlebell squats correctly (i.e., maintain squat depth and BPM). The hypothetical 1-RM was calculated according to Epley’s equation ($1RM=\left[\left(0.033*reps\right)*execution weight\right]+execution weight$) [61] with the number of repetition the final weight was lifted in the last set. The mean values and standard deviations of the sample characteristics collected during the pretesting session can be taken from Table 1.

**Table 1:** Mean values and standard deviations (*SD*) of the sample’s characteristics

	Young Adults (*n* = 18)	
Parameters	*Mean*	(*SD*)
Height [cm]	173.1	(9.3)
Body weight [kg]	69.6	(14.5)
MoCA Score	27.9	(1.4)
Exercise amount [min/week]	377	(183)
PA in leisure time [min/week]	444	(466)
1-RM [kg]	40.4	(13.3)
1-RM normalized to body weight	0.6	(0.2)
Self-rated experience in RT [1-10]	7.4	(1.9)

1-RM = calculated one-repetition maximum, MoCA = Montreal Cognitive Assessment, PA = physical activity, RT = resistance training

### Testing session

At the beginning of the testing session, participants were asked to rate their current mood on the feeling scale. Participants then did a five-minute general warm-up on a cross-trainer (1 W per kilogram of body weight) prior to a set of ten squats in each ground condition following the individual repetition velocity for a specific warm-up and familiarization.

As changing light conditions evoke pupils to dilate or constrict according to the pupillary light reflex [62], ceiling lights were turned on and the room was darkened with blinds ensuring standardized light conditions for the measurement of pupil size. Pupil diameter was recorded using Eye Tracking Glasses (ETG) from SensoMotoric Instruments (SMI). Two eye cameras located at the bottom of the SMI ETG recorded eye movements and changes in pupil size at a frequency of 60 Hz (120 Hz binocular). In addition, the scene camera (field of view: 60° horizontal, 46° vertical) recorded the participants’ view. Clear infrared filtering plastic lenses were inserted into the frame of the ETG according to the manufacturer’s specifications. A laptop (Lenovo ThinkPad X230) was connected to the ETG via a USB cable. Pupil diameter was recorded with iViewETG software (version 2.7). The ETGs were individually adjusted by fitting a narrower or wider nose bridge to ensure a secure and stable fit. The headband and cables of the SMI ETG were attached to the participant’s back.

Prior to data collection, a three-point calibration of the SMI ETG was performed following manufacture guidelines. For this purpose, three black adhesive tape markings were attached to a white wall in a triangle shape, which participants had to fixate according to the provided instructions. After calibration, the adhesive marks were removed from the wall. Data collection started with a 10 s baseline measurement. For this, participants were instructed to stand relaxed while looking at the wall about five meters away without fixating on a specific point and

keeping their head straight throughout. Additionally, they were told to blink as little as possible while measuring. After the baseline measurement, participants were prepared for the squatting exercise. The squatting exercise consisted of a pre-phase, squatting phase and a post-phase and was conducted with an additional weight of approx. 40% 1-RM according to Herold et al. [27]. As the kettlebells were only available at intervals of four kilograms, the 40% of the 1-RM was rounded up or down. For the pre-phase, participants were instructed to hold the additional weight at chest height for about ten seconds in a standing position with the gaze at the wall. Following the 10-seconds pre-phase, the metronome sound indicated that participants had to start squatting for ten repetitions. They were instructed to reach the lowest point of the squat until the second sound. In contrast to the sets in the pretesting session, the experimenter did not announce the last of the ten repetitions. Instead, the participants completed the set after the metronome had stopped. After finishing the set of squats, participants held the standing position again for another ten seconds with the weight in front of their chest. After completing the squatting exercise, participants rated their overall perceived exertion (RPE) on the Borg scale using a single number, which ranged from 6 to 20 and included both physical and cognitive exertion. A rating of 6 indicated no exertion at all, while a rating of 20 indicated maximum exertion [63].

Participants were told to avoid fixating a point while squatting, as pilot measurements with fixation of a (larger) point have shown fluctuations in the recording of pupil diameter. Additionally, they were also asked to blink as little as possible in order to avoid occlusion of the eye and missing pupil data. Furthermore, they should refrain from counting repetitions as this may imposean additional cognitive demand. On the kettlebell, the Vmaxpro sensor was attached to record the number of repetitions, the vertical displacement and the movement velocity while squatting. Annotations were set manually at the start and at the end of the squatting phase. The sequence was performed once on the even floor and once on the BOSU® Balance Trainer. There was a break of at least three minutes between the two ground conditions. The order of conditions (stable, unstable) was counterbalanced among the participants.

In order to internalize the measurement procedure, a practice run was completed prior to data collection. After each set of squats, the three-point calibration was repeated if necessary. The squatting exercise was repeated if participants failed to accomplish the set of squats (e.g., due to balance loss). The squatting exercise lasted an average of 24 s in each condition (stable: M = 23.7, SD = 4.7s; unstable: M = 23.7, SD = 4.1s).

### Data processing

In processing the data, we followed standard processing recommendations [64, 65]. SMI software BeGaze™ 3.7 was used to analyze the pupillometric data. Pupil diameter of the left and right eye including events such as blinks and eye movements were exported and further analyzed using a custom written MATLAB script (Version R2022b, Math-Works, Inc.). First, the pupil data was reduced by identifying and rejecting blinks. Since the measurement of pupil size is distorted by the closing and opening of the eyelid [62], pupil data at which the BeGaze™ 3.7 software automatically detected a blink were removed and linearly interpolated [48]. Additionally, samples that deviated visually from the trend line and showed a disproportionate large change in absolute pupil size [65] were assumed to contain noise and artifacts. We identified these samples (partially caused by saccades) in each condition using Matlab’s outlier identification function. Outliers were defined as values that were more than three scaled median absolute deviations away from the median [66]. These values were removed and the remaining data was linearly interpolated [48]. For data quality purposes, the maximum proportion of replaced samples in the baseline measurement and squat exercise trials was set to 40%, following the methodology of Saeedpour-Parizi et al. [48]. Data sets exceeding 40% were excluded from further analysis. In our study, the data of all participants could be used. The mean percentage of excluded samples per condition was: baseline = 7% (*SD* = 16), stable condition = 5% (*SD* = 3), unstable condition = 3% (*SD* = 3). After removing blinks and artifacts, the pupil data was filtered using a 3-point moving average digital filter, resulting in an effective 8.8 Hz filter [64]. Due to the perspective of the cameras located at the bottom of the SMI ETG, pupil will take on an elliptical shape if eyes move upwards. In order to compensate for this disturbing factor, the pupil data of the right and left eye were averaged at each time point in each condition [48]. Phases of the squatting exercises (pre, squatting, post) were separated by using the annotations manually set during the measurement. For the compensation of random fluctuations and increasing statistical significance, the pupil data recorded during squatting exercise were normalized using a baseline correction [62]. Following Mathôt [62], who advocated a short baseline period, and White and French [49], who determined the baseline pupil diameter in the first three seconds, we calculated the baseline pupil diameter in the second to third second (60 frames). We excluded the first second for the baseline pupil diameter, as pilot measurements showed that they contained fluctuations that led to bias in the baseline-corrected data. As recommended by Mathôt [62], a subtractive baseline correction (corrected pupil diameter = pupil diameter - baseline) was chosen, because it is less susceptible to distortion (e.g., due to unrealistically small pupil diameter caused by blinking or data loss) compared to a divisive baseline correction (corrected pupil diameter = pupil diameter/baseline).

### Statistical analysis

A mean value was calculated for the processed and baseline corrected pupil size data (mean PD) during squatting exercise. Additionally, we calculated the mean value for the pre squat phase, during which participants hold the weight in a standing starting position, representing the baseline data for each condition. Similar to the baseline measurement, we used the 2nd to 3rd second to calculate the mean value for the pre phase. Squatting performance was assessed by the movement velocity and vertical displacement of the kettlebell measured by the Vmaxpro sensor. Mean PD, movement velocity, vertical displacement and RPE for stable and unstable ground conditions were checked for normal distribution by the Shapiro-Wilk test and compared by a paired student t-test between conditions. Further, we conducted Bayesian t-tests and calculated the Bayes Factors (*BF*_10_) to enhance the explanatory power of the inference t-tests results. The Bayesian t-test used a default uninformed Cauchy prior width of 0.707, as recommended by Wagenmakers et al. [67]. We interpreted *BF*_10_ as follows: 1 < anecdotal ≤ 3; 3 < moderate ≤ 10; 10 < strong ≤ 30; 30 < very strong ≤ 100; extreme > 100 [68]. The criteria for evidence supporting the alternative hypothesis (H1) was set as *BF*_10_ > 3, while evidence supporting the null hypothesis (H0) was set as *BF*_10_ < 0.33.

Significant differences between conditions were checked by a non-parametric Wilcoxon tests in cases of violation of normality. In addition, we calculated effect sizes (Cohen’s d) with d-values ≤ 0.49 indicating small effects, 0.50 ≤ d ≤ 0.79 indicating medium effects, and d ≥ 0.80 indicating large effects [69]. All statistical calculations were performed using JASP (version 0.16.4). The significance level was set at α = 0.05 for all statistical analyses.

## Results

All data were normally distributed except the RPE for the unstable condition. Therefore, we used a non-parametric test to compare RPE between conditions.

### Pupil diameter

The baseline data for all conditions are represented by the baseline measurement (standing only) and the pre squatting phases (holding the weight in a standing position on a stable or unstable surface). During standing (referred to as baseline), the participants had an average pupil diameter of 3.93 mm (*SD* = 0.57). On average, the pupil diameter was slightly larger during the pre phase of both stable (*M* = 4.24, *SD* = 0.57 mm) and unstable (*M* = 4.35, *SD* = 0.60 mm) conditions.

**Fig. 2:**
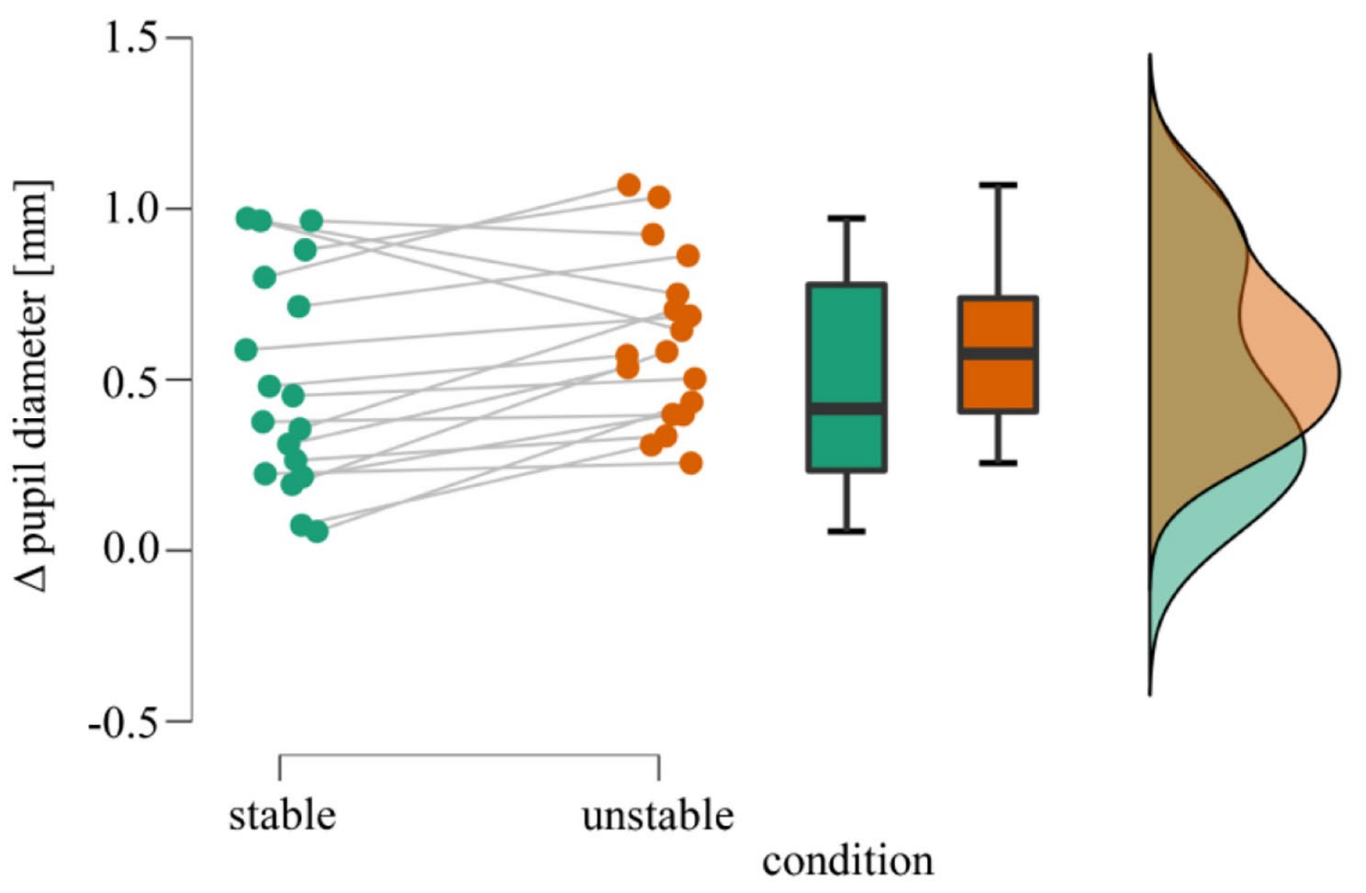
Raincloud plot of the mean pupil data during squatting on stable and unstable ground conditions

Pupil dilation relative to the baseline during kettlebell squats in stable and unstable ground conditions is shown in Fig. 2. Student’s t-test revealed that pupil size was significant larger while squatting in unstable ground (*M* = 0.61, *SD* = 0.25 mm) compared to stable ground conditions (*M* = 0.49, *SD* = 0.32 mm), *t* (17) = -2.63, *p* =.018, Cohen’s *d*_Z_ = -0.62, with moderate evidence in favor of H1 (*BF*_10_ = 3.13). Additionally, both conditions significantly differed from 0 (stable: *t* (17) = 6.62, *p* <.001, Cohen’s *d*_Z_ = 1.56; unstable: *t* (17) = 10.57, *p* <.001, Cohen’s *d*_Z_ = 2.49) with extreme evidence for larger pupil size while squatting compared to standing (*BF*_10_ stable: 4725.41; *BF*_10_ unstable: 1.75 * 10^6^).

### Rating of perceived exertion

**Fig. 3:**
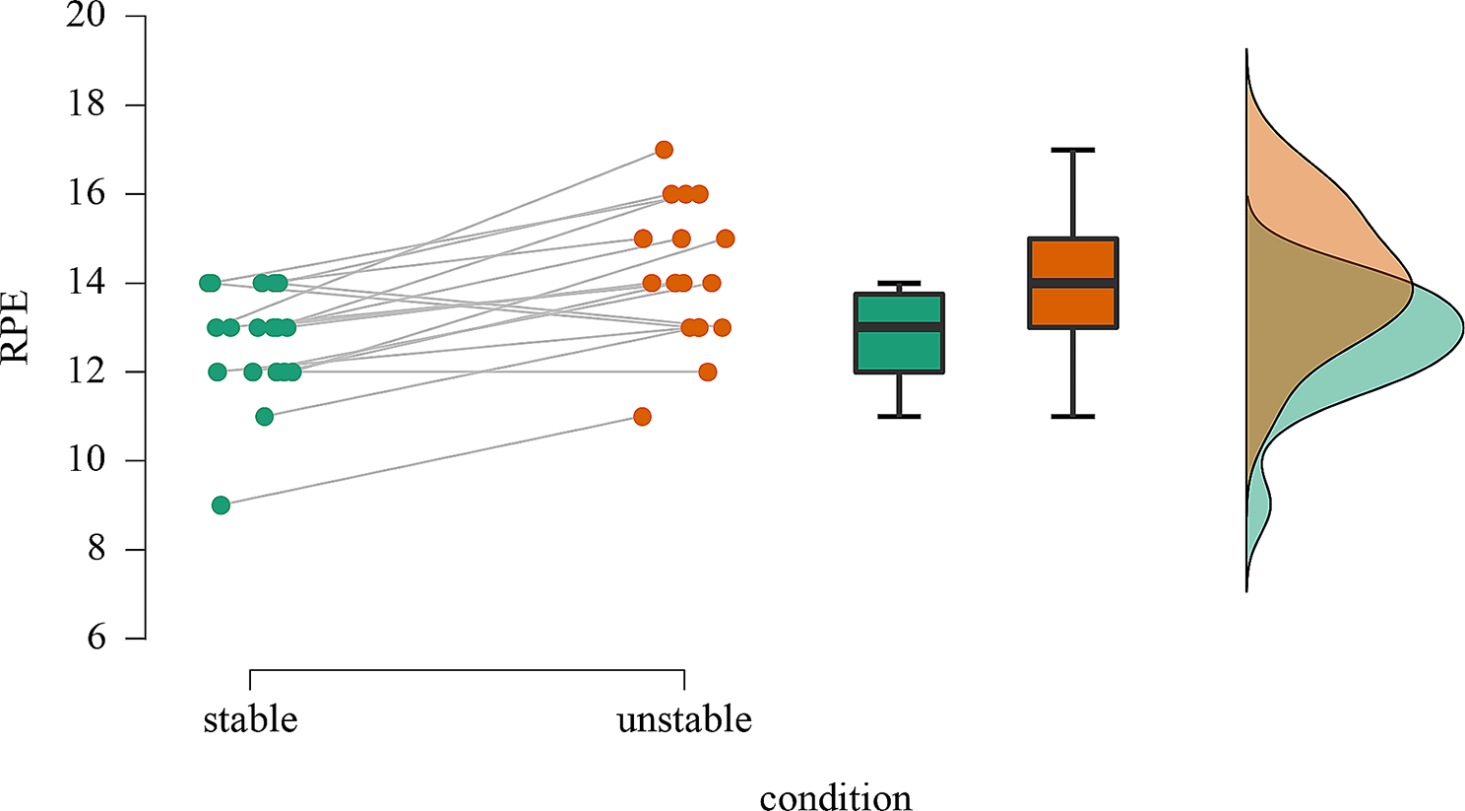
Raincloud plot of the rating of perceived exertion (RPE) in stable and unstable ground conditions

Subjectively rated exertion on the Borg scale was found significantly higher for squatting with surface instability (*M* = 14.2, *SD* = 1.5) compared to stable ground conditions (*M* = 12.7, *SD* = 1.3), *z* = -3.24, *p* <.001 (see Fig. 3). Bayes factor indicated extreme evidence in favor of H1 (*BF*_10_ = 349.30).

### Squatting exercise

Although squatting exercise were performed in accordance to an individual repetition velocity equal for both conditions, movement velocity, *t* (17) = 5.31, *p* <.001, Cohen’s *d*_Z_ = 1.25, and vertical displacement, *t* (17) = 2.94, *p* =.009, Cohen’s *d*_Z_ = 0.69, significantly differed between stable and unstable ground conditions. Participants performed squats on stable ground significantly faster and deeper than on the unstable device. Mean values of the performance parameters are presented in Table 2.

**Table 2:** Mean values and standard deviations (*SD*) of the squatting exercise

	stable		unstable			
Parameters	*Mean*	(*SD*)	*Mean*	(*SD*)	*p*-value	*BF*_10_
Movement velocity [m/s]	0.59	0.12	0.54	0.11	< 0.001	458.67
Vertical displacement [m]	0.59	0.05	0.57	0.05	0.009	5.70

## Discussion

The aim of this study was to investigate the cognitive activation during acute resistance exercise on stable and unstable surfaces measured by pupil size. The results show that the pupils dilated significantly, when executing squats compared to standing upright, and that squats on an unstable surface resulted in a significantly greater pupil diameter than squats on stable ground. The increase in pupil size compared to standing (baseline) indicates that squats with free weights lead to cognitive activation and an increased level of arousal. The significant difference in pupil size between conditions (stable vs. unstable) supports our hypothesis that unstable devices used during resistance exercise can further increase effort and acute cognitive activation while exercising. This increase in cognitive activation may be related to increased metabolic [54] as well as coordinative [8, 10] and perceptual demands [9] associated with the use of unstable devices.

Since pupil size is linked to the activation of neuromodulatory systems, including the LC-NE system, it seems reasonableto assume that using unstable devices while exercising leads to a greater release of norepinephrine in the brain. In addition to regulating muscle tone and contributing to postural control [70], the release of norepinephrine promotes information processing and attentional focus by facilitating sensory gating and filtering in different brain regions, including the frontal areas related to cognitive and executive functions (EFs) [38, 40, 43]. Since norepinephrine also releases into frontal areas of the brain, the observed increase in pupil size during squats on stable and unstable surfaces could indicate the activation of neuronal circuitry associated with EF tasks as suggested by the cognitive stimulation hypothesis [15].

Due to the limited insight into the brain provided by pupillometry, we can only speculate about the cortical mechanisms associated with pupil dilation. However, the following section discusses various mechanisms that could activate the reticular activating system.

### Possible mechanisms for the activation of the reticular activation system

As discussed by Amico et al. [71], muscle contractions while squatting may lead to the activation of the LC via the *muscle spindle pathway*. During muscle contraction, muscle spindles become active and send action potentials to the brainstem. These ascending signals ensure activation of the LC [71, 72].

In addition to muscle spindle activity, mechanosensitive and metabosensitive receptors in active muscles (e.g., also the heart and lungs) as well as arterial baroreceptors send information (e.g., about blood pressure) to the nuclei in the brainstem, resulting in activation of the sympathetic system and release of NE [73–75]. These mechanisms are referred to as the *mechanoreflex*, *metaboreflex*, or *baroreflex* [74, 76, 77].

The *vagus nerve pathway* or *catecholamine hypothesis* describes the indirect activation of the LC and the reticular activating system via activation of the vagus nerve during moderate-intensity exercise in an intensity-dependent manner [73]. The increase of peripherally circulating catecholamines (e.g., epinephrine, norepinephrine, dopamine) during exercise leads to the activation of β-adrenoceptors on the vagus nerve. Further, the vagus nerve innervates the nucleus tractus solitarii (NTS) in the brainstem which projects onto the LC [72, 73].

A third possible mechanism describes the activation of the LC as an effect of processes of cognitive attention. The execution of squats is thought to act as a goal-directed behavior with action goal planning in frontal and parietal areas. The action goal is send and controlled via top-down processes. This activity in frontal and parietal structures leads to the activation of the LC via top-down processes, resulting in pupil dilation [72, 78]. Overall, it can be stated that the execution of kettlebell squats can activate the LC-NE system via different pathways with the consequence of increased activation of further parts of the central nervous system in addition to pupil dilation.

### Pupil size measures in postural tasks and exercise

Some studies have used pupillometry to assess effort and autonomic activation in motor tasks [46–49], resistance exercises [77, 79] and postural control tasks [50, 51]. In detail, muscle contraction during a submaximal hand force task was found to lead to pupil dilation [77]. Their results support the metaboreflex/ mechanoreflex or muscle spindle pathway as a possible cause of LC-NE system activation and pupil dilation. Mather et al. [79] showed that a maximal handgrip task activated the LC-NE system as measured by pupil dilation in young and older women, allowing participants to benefit from cognitive activation in the subsequent cognitive task.

Kahya et al. [51] found that young adults showed greater cognitive activity when postural demands were increased by visual occlusion during upright standing. As a consequence, they assume that visual occlusion increases cognitive demands because additional neural resources are required to maintain balance [51]. Compared to our study, the large effect size of visual occlusion (Cohen’s dz = -4.5) suggests that it has a considerable effect on cognitive activation. However, Kahya et al. [51] calculated an index of cognitive activity to assess mental effort rather than the absolute change in pupil diameter as their study involved changing light conditions due to visual occlusion. As a result, the effect sizes of changing visual perception in their study cannot be compared with the effect size of changing ground condition in our study. In another study, Kahya et al. [50] found that cognitive activation increased with increasing task difficulty through visual occlusion and an additional cognitive task while standing. In addition, they examined fronto-central alpha power as another indicator of task difficulty and sensorimotor information processing [80]. By using electroencephalography (EEG), they found a strong negative correlation between pupil dilation and fronto-central EEG alpha power while standing with eyes closed. This finding is consistent with previous studies that showed a decrease in fronto-central alpha power with increasing balance demands [80–82]. However, fronto-central alpha power increased significantly when visual information was removed for postural control. The authors explained this by suggesting that alpha power is involved in sensory processing and appears to increase not only in the occipital lobe but also in other brain regions while the eyes are closed. Other studies investigating changes in cognitive activation during postural tasks by using neurophysiological measures such as EEG or functional near infrared spectroscopy (fNIRS) found increased activation in sensorimotor brain areas with increasing balance demands [10, 83].

Further studies also indicate a relationship between exercise intensity and pupil size [32, 52, 53, 84]. While some studies examined the effects of graded aerobic exercise on pupil size [52, 53, 84], others examined the effects of a handgrip task with increasing force output [32]. While Hayashi et al. [53] showed that pupil diameter increased with increasing exercise intensity, a recent study found pupil dilating at even low physical intensity which was interpreted with the activation of the reticular activation system [52]. Both findings support the catecholamine hypothesis that exercise affects pupil size in an intensity-dependent manner. Zénon et al. [32] showed that the pupil responded with an increase in diameter with raising handgrip force intensity and perceived exertion. Based on their findings, they suggested that pupil size reacts to both cognitive and physical exertion, because cognitive and physical exertion lead to similar cardiovascular and catecholamine responses as well as to the activation of the autonomic nervous system.

In our study, although heart rate (HR) was not measured as an objective parameter of physical exertion, the significantly higher RPE suggests that participants felt more exerted when performing squats on unstable surface. Converting the mean RPE of both conditions to HR (HR = RPE*10, [85]) would yield a mean HR of 127 for the stable condition and 142 for the unstable condition. As Hayashi et al. [53] found a significant difference in pupil size between HR categories of 120–140 and > 140, it can be argued that the different perceived intensities could account for the difference in pupil size between stable and unstable ground conditions.

However, our study found that squatting on stable and unstable surfaces resulted in pupil dilation, suggesting cognitive activation and arousal. This effect was further increased due to surface instability. It remains unclear, which mechanism(s) trigger the cognitive activation while squatting. Future studies could combine pupillometry with neurophysiological (e.g. EEG, fNIRS), neuromuscular (e.g. electromyography (EMG)) and neurochemical measurements during squatting to (1) investigate the relationship between pupil diameter, brain activity, and physiological biomarkers and (2) provide a temporal and spatial resolution of brain activity and thus a further perspective on cognitive activation while squatting with different balance demands.

Furthermore, the impact of metastable resistance exercise on cognitive function is not yet clear. Kuwamizu et al. [86] investigated the acute effects of very-light-intensity exercise and found an increase in pupil size during exercise as well as prefrontal cortical activation (assessed using fNIRS), and improved performance in an EF task (Stroop task) after the acute bout of exercise. Additionally, they observed that the degree of change in pupil dilation correlated with the degree of improvement in Stroop performance. In our study, we did not measure cognitive task performance. Therefore, it remains unclear, whether pupil dilation during metastable resistance exercise is associated with enhanced executive task performance. To address this knowledge gap, it is a promising area for further research to assess whether cognitive performance (e.g., EF) and pupil dilation changes after a single session of resistance exercise on stable and/or unstable surfaces and whether changes in pupil diameter during resistance exercise can predict exercise-induced changes in cognitive performance (e.g., EF). The results could provide additional insights into the cognitive processes that occur during and after resistance exercise which, in turn, can foster our understanding of the mechanisms of exercise-induced changes of executive performance.

The plot of individual data points in Fig. 2 indicates individual pupillary responses to kettlebell squats maybe due to inter-individual differences in arousal. Individually different pupillary responses were also found for graded exercise and handgrip force [32, 52]. Pupillary response is generally influenced by subject variables such as medical conditions (e.g., retinal disease), age, medication and drugs (e.g., opioids, cocaine), or sleepiness [64]. During the pretesting session, we inquired about medical conditions, medication, and drug use. Since none of the participants reported using any of the mentioned substances, it seems unlikely that such factors have influenced our findings. Instead, the varying levels of experience in resistance training among the participants may have led to different reactions of the pupil. While not directly comparable to motor task experience, research has shown that individuals’ intelligence scores correlate with the amplitude of the pupillary response during mental arithmetic tasks [44, 87], suggesting that individuals with more experience or skills are expected to require less effort to complete a cognitive task [38]. However, it is unclear whether this correlation reflects differences in resource allocation or processing efficiency [44, 64]. Another possible explanation for inter-individual differences in pupillary response to the changing ground conditions could be a low responsiveness to stressors [86, 88].

### Limitations

One limitation of the study is that we set a fixed repetition velocitiy for the execution of squats and averaged the repetition velocity of the stable and unstable conditions. Although the repetition velocity was individually determined, it could have put time pressure on the participants. Despite having the same set of repetition velocity for both conditions, the execution of squats differed in movement velocity and vertical displacement with higher values in the stable condition. This finding suggests that participants preferred to execute kettlebell squats at a slower pace on unstable ground. The results should be interpreted with caution because time pressure may have caused stress, leading to an increase in pupil size, particularly on the unstable surface. Another limitation of this study is the absence of a measure of environmental light conditions (i.e., illumination) to enable comparisons of pupil size and dilation with other studies in the field [52, 86].

### Conclusion

This is the first study to measure pupil dilation during an acute bout of resistance exercise on stable and unstable surfaces. Our results indicate that kettlebell squats on an unstable device lead to a higher pupil size which may indicate a higher activation of specific neuromodulatory systems such as the LC-NE system. Therefore, it is assumed that people require more physical and mental effort to perform resistance exercises safely (i.e. with correct technique and without loss of balance) with surface instability. Resistance training with and without unstable devices can be recommended to people of all ages as a physically and cognitively challenging training program that can contribute to the preservation of both physical and cognitive functions.

## Data Availability

The datasets generated during and/or analyzed during the current study are available from the corresponding author on reasonable request.

## Abbreviations

1-RM: one-repetition maximum
BDNF: brain-derived neurotrophic factor
BF: Bayes factor
BPM: beats per minute
BSA: Bewegungs- und Sportaktivitätsfragebogen
EEG: electroencephalography
EF: executive functions
EMG: electromyography
ETG: eye tracking glasses
fNIRS: functional near infrared spectroscopy
HR: heart rate
IGF-1: insulin-like growth factor-1
IMU: inertial measurement unit
LC: locus coeruleus
M-RM: multiple repetition maximum
MoCA: Montreal Cognitive Assessment
NE: norepinephrine
NTS: nucleus tractus solitarii
PAR-Q: Physical Activity Readiness Questionnaire
PD: pupil size data
RIR: repetitions in reserve
RPE: rating of perceived exertion
SMI: SensoMotoric Instruments
TuT: time under tension
